# Shame and guilt in the suicidality related to traumatic events: A systematic literature review

**DOI:** 10.3389/fpsyt.2022.951632

**Published:** 2022-09-30

**Authors:** Flavie Ollivier, Andréa Soubelet, Stéphane Duhem, Susanne Thümmler

**Affiliations:** ^1^CoBTek, FRIS, Université Côte d'Azur, Nice, France; ^2^Service Universitaire de Psychiatrie de l'Enfant et de l'Adolescent, Hôpitaux Pédiatriques de Nice CHU-Lenval, Nice, France; ^3^Service de Psychiatrie, CRP HdF, CHU Lille, Lille, France; ^4^Department of Psychiatry, CHU Lille, Lille, France; ^5^F2RSM Fédération Régionale de Recherche en Santé Mentale et Psychiatrie, Lille, France; ^6^Inserm, Centre d'Investigation Clinique, CHU Lille, Lille, France

**Keywords:** shame, guilt, suicidal thoughts, suicide attempt, suicide, psychotrauma, posttraumatic stress disorder (PTSD)

## Abstract

**Background:**

Shame and guilt are involved in suicidality and in post-traumatic stress disorder. However, few studies have explored the implication of those emotions in the suicidality of patients exposed to traumatic events.

**Objective:**

The objective of this literature review was to examine the implication of shame and guilt in the suicidality of individuals who have experienced potentially traumatic events or been diagnosed with post-traumatic stress disorder. These two emotions are part of post-traumatic stress disorder and suicidality. Moreover, when individuals perceive that their coping strategies are inadequate, they may view suicide as a relief from suffering.

**Method:**

This review was conducted according to PRISMA method. We used combinations of search words for traumatization, suicide ideation and behavior and shame and guilt to search for empirical studies in common databases in psychology and medicine.

**Results:**

Among 137 identified articles, 9 full texts were retained. Results suggest that shame and guilt were involved in all aspects of suicidality in patients who had experienced traumatic events or been diagnosed with post-traumatic stress disorder. The degree of shame and guilt differed with the type of traumatic event, notably affecting individuals who had experienced military combat, physical or sexual abuse, or emotional or physical neglect.

**Conclusion:**

Shame and guilt are implicated in suicide's risk. Future research is now needed to determine whether greater attention to these two emotions would enhance our understanding and anticipation of suicidal behavior in those who have experienced a potentially traumatic event or been diagnosed with post-traumatic stress disorder.

## Highlights

- Shame and guilt may be risk factors for suicidality in patients who had experienced traumatic events or been diagnosed with post-traumatic stress disorder.
- Effects of shame and guilt on the intensity and frequency of suicidal ideations vary depending on the type of trauma.

## Introduction

Suicidality refers to Suicidal Ideation (SI) and suicidal behavior, whether lethal or not, and their frequency (1). According to the World Health Organization (2), about 800,000 people die by suicide every year worldwide. In France, 9,000 people die by suicide annually (3), and in 2019, this was three times the number of deaths linked to road accidents (4). Also in 2019 in France, 5% of all individuals between 18 and 75 years old reported SI within the past 12 months, and 7% had made a Suicide Attempt (SA) in their lifetime (3).

A suicidal crisis can be caused by a potentially traumatic event (5, 6). Individuals exposed to actual death, threat of death, serious injury, or sexual violence, against self or others, are likely to develop symptoms of acute stress, as defined in DSM-5 (7): a negative mood and intrusive, dissociative, avoidance and arousal symptoms. If symptoms persist for more than a month, Post-Traumatic Stress Disorder (PTSD) is diagnosed (7). In a study of 94 patients with chronic PTSD, Tarrier and Gregg (8) found that 56.4% reported at least one aspect of suicidality since the traumatic event, much higher than that for the general population.

The experience of a traumatic event induces a change in causal attributions, which is linked to the development of PTSD symptoms (9, 10) and negative emotions. Causal attributions refer to how individuals use information about an event and their perception of their abilities and feelings to explain the event (11). The experience of a traumatic event may cause feelings of helplessness in the victim, thereby prompting the search for meaning in the situation to regain a sense of control (10). Especially, shame and guilt are negative emotions which can be developed after a traumatic event. These emotions are often combined (12) and are linked with PTSD severity (12).

According to Tangney and Dearing (13), shame and guilt have an influence on the behavior, and then directly impact our interpersonal sphere, but also how we perceive ourselves. In fact, shame and guilt are related to self-awareness and are part of self-assessment and introspection (14). Shame reflects a negative intrapersonal and global assessment of oneself, which causes individuals to want to hide from others. Guilt is the negative evaluation of their actions or behaviors and results in specific regrets or remorse.

The model of Joseph, Williams and Yule (10) proposed a link between PTSD, negative emotions and the interpretation of causes, issues, and consequences of a traumatic experience. According to this model, when individuals proceed to internal attributions following an event but do not perceive a sense of control, they develop shame. On the other hand, if they perceive control, they develop guilt.

Shame and guilt are involved in suicidality. Whether it be the suicidal crisis model (15), psychiatric models (16, 17) or interpersonal suicide theory (18), all highlight the importance of emotions, such as shame and guilt, in suicidality.

The suicidal crisis model (15) suggests that individuals who perceive only inadequate solutions and coping strategies may come to think of suicide as a means of alleviating their suffering. According to this model, someone in a suicidal crisis is overwhelmed with emotions and the feeling of helplessness.

The interpersonal theory of suicide (18) defines more precisely the implication of shame and guilt in the suicidality. According to this theory, guilt has an interpersonal dimension (19). This theory is based on the following observation: social isolation is one of the strongest predictors of SI, SA, and death by suicide. For example, when the need for belonging is unmet, feelings of isolation and of being disconnected from others are strengthened and SI may appear. Also, the discomfort experienced when individuals perceive themselves as a burden to others may give rise to self-hatred and the thought that they have so many failings that others are forced to be responsible for them. When the perception of being a burden to others and the sense of not belonging anywhere are combined with hopelessness, individuals do not perceive the possibility of positive change, which causes active SI and a potential SA.

The psychiatric models have been able to show the impact of diagnoses like depression on suicidality in various populations (20–25). According to Hegerl (16), depression is a risk factor for SA and death by suicide because the perceptions of reality are distorted by the psychiatric disorder: individuals see no hope and experience their suffering as unbearable. Notably, depressive symptoms are closely linked to shame and guilt, regardless of age and gender (17). Moreover, a meta-analysis by Krysinska and Lester (26) showed that the relationship between PTSD and suicidality is particularly impacted by comorbid depression and psychiatric history before the traumatic event.

Despite the clear links between shame, guilt, and suicidality, little is known about how shame and guilt are implicated in the suicidality of patients diagnosed with PTSD. This systematic literature review thus examined potential links between shame, guilt, and suicidality in the context of a PTSD diagnosis to enrich our understanding and improve our evaluation of negative emotions in the suicidality of PTSD patients.

## Method

A systematic literature review was carried out following PRISMA criteria. The following databases were searched: PUBMED, PSYCINFO, Cochrane, Wiley Online Library and Scopus.

Search terms were: (shame) AND (guilty OR guilt) AND (“suicidal ideation” OR suicide OR suicidality OR “suicide attempt”) AND (PTSD OR “posttraumatic stress disorder” OR “post-traumatic stress disorder” OR “traumatic stress” OR “post traumatic symptoms” OR “traumatic stress symptoms” OR trauma). It was chosen to limit the research to articles containing both shame AND (guilty or guilt) because shame and guilt are correlated (27, 28) and the aim was to compare both to determine which one predicts the most suicidality in patients involved in traumatic events.

### Inclusion and exclusion criterion

**Figure 1:**
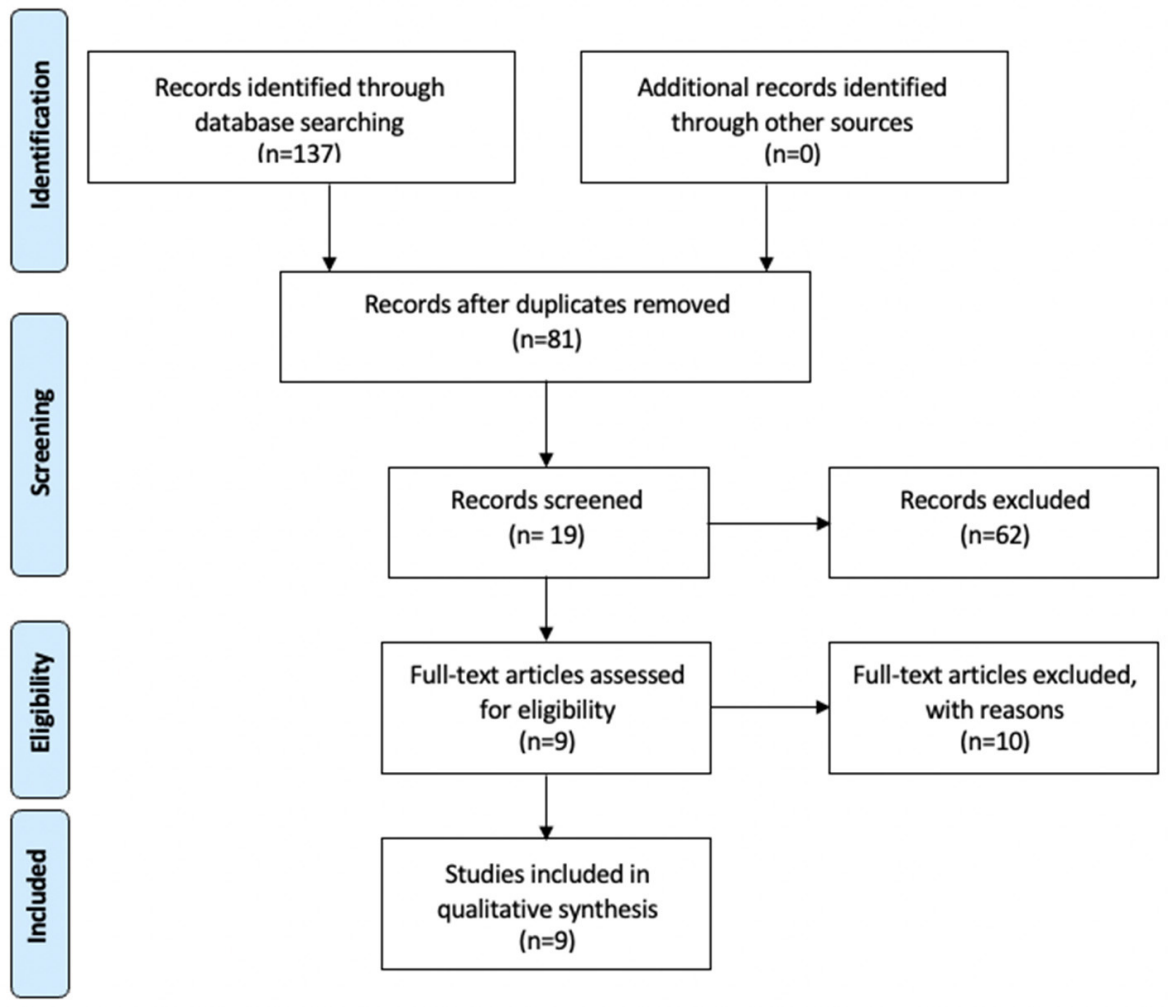
PRISMA flow diagram.

The data were extracted between January and May 2021 (see Figure 1). After removing duplicates, 81 articles were recorded. 62 were excluded after analysis of titles and abstracts following the inclusion and exclusion criteria presented in Table 1. Over the 19 remaining, 10 were excluded after full-text analysis. Exclusions were based on the following reasons: methodology was not well described in two articles, four articles only studied shame or guilt but not both, and 4 others were not specifically studying the link between shame, guilt, suicidality and PTSD. Finally, 9 articles were included in the qualitative synthesis (see Supplementary Table 1).

**Table 1:** Criteria of inclusion and exclusion.

**Inclusion criteria**	**Exclusion criteria**
• Studies published in English	• Case studies, multiple case studies, theses, book chapters, books, clinical trial protocols
• Studies mentioning shame, guilt, suicide and PTSD in the title and/or abstract	• Full text unavailable
• Direct measures of suicidality	• Methodology insufficiently described
• Evaluation of the experience of a potentially traumatic event and evaluation of PTSD symptoms	• Tools for measuring shame and guilt not standardized
	• Studies focused on peri-traumatic and acute phases

### Statistical tests used in the articles included

Correlations used in the articles were intercorrelations (29, 30), zero order correlations (27, 31), partial correlations (28, 31), Spearman correlation (32) and Pearson correlation (28). T-test was also used, to examine differences between women victims of abuse and women not concerned by abuses (28) and to compare assessment pre- and post-treatment (33).

Some papers investigated mediation processes between shame and guilt and SI with path analysis (29, 30), linear regression (27), calculation of indirect effects (32), Hayes (34) method (31), and partial mediation (29).

Path analyses were conducted (32) to study the mediation of the type of trauma in the relation between guilt and shame and SI.

The interaction between childhood sexual abuse and guilt and shame was studied using hierarchical regression analysis (28).

Some also conducted analysis of variance to compare mean guilt and shame scores according to history of SI (27).

Other data analyses were conducted using hierarchical linear models (33, 35), mixed models of variance (33), MANOVA (35), Wonnacott and Wonnacott's (36) procedure (29) and Preacher and Hayes' procedure (29).

## Results

One hundred and thirty-seven studies were retrieved from the databases and 9 were retained for this systematic review (see Figure 1). Of these nine (Supplementary Table 1), three (27, 29, 30) showed an association between suicidality and PTSD, which was even higher for patients diagnosed with borderline personality (33, 35). Three articles (31, 32, 37) did not investigate this association and one study of women hospitalized in psychiatric clinics (28) reported that the frequency of SI was lower in the women who had been sexually abused in childhood than in those who had not been abused.

### Involvement of shame and guilt in exposition to a potentially traumatic event or PTSD

Five studies (27, 29–31, 33, 35) highlighted the involvement of both shame and guilt in patients with PTSD or those who had been exposed to potentially traumatic events. Three articles (33, 35, 37) did not explore this link. One study (28) found a positive and significant association between shame and sexual abuse, but the association was negative with physical abuse. Sekowski et al. (32) found significant associations between shame and emotional abuse, emotional neglect, and physical neglect, but not physical or sexual abuse. This same study showed a significant association between guilt and emotional abuse, emotional neglect, and physical neglect and sexual abuse, but not physical abuse.

### Involvement of shame and guilt in the suicidality of patients exposed to potentially traumatic events or diagnosed with PTSD

Five out of the nine studies (28, 30–32, 37) showed implication of shame and guilt in the suicidality of patients who had experienced potentially traumatic events or been diagnosed with PTSD in several populations, notably adolescent girls who had been sexually abused (29, 30), soldiers receiving psychiatric care (27), soldiers deployed in Afghanistan or Iraq (31). Among 106 women who had been sexually abused in childhood (37), those with high scores on a scale measuring negative emotions were more likely to have SI.

However, the association of shame, guilt, and suicidality in the context of PTSD was not demonstrated in one population: adolescents who had experienced sexual violence (29). In that population, 45.20% of the variance in PTSD symptoms was explained by shame and self-blame. The variance in SI was best explained by self-blame, shame, and depressive symptoms when PTSD symptoms were not included in the analysis. This indicates that self-blame, shame, and depressive symptoms might better explain SI than PTSD symptoms in this population. Also, 41.60% of the variance in SI was explained by self-blame, shame, and depressive symptoms, but only 1.08% of the variance in SI was explained by symptoms of PTSD.

### Influence of the type of traumatic event on SI, shame, and guilt

The results of this review also highlighted that the type of trauma influences the relationship between the frequency and intensity of SI and shame and guilt (28, 32). Sekowski et al. (32) differentiated the implication of shame and guilt in SI based on the type of trauma: physical abuse, physical neglect, sexual abuse, emotional abuse, and emotional neglect. Emotional abuse, emotional neglect, and physical neglect were significantly correlated with shame and guilt. Sexual abuse and physical neglect were indirectly related to the intensity and severity of SI through guilt and depressive symptoms.

Conversely, in Kealy et al.'s study (28), higher levels of guilt appeared to be positively associated with more frequent SI only in women who had not been sexually abused as children. Nonetheless, the authors reported that stronger feelings of shame appeared to be positively related to an increased frequency of SI in women who had been sexually abused during childhood. On the contrary, in the study of Sekowski et al. (32), shame did not appear to be a mediating variable in the relationship between abuse and the intensity and severity of SI.

Furthermore, in a population of soldiers who served in Afghanistan and Iraq (31), acts of omission—i.e., situations in which soldiers witnessed violence and those in which they believed they might have acted to prevent the violence—and acts of commission were significantly associated with shame and guilt. Only omitted acts were significantly and strongly associated with suicidality and PTSD. The relationships between omission, suicidality and PTSD were mediated by altered perceptions—i.e., a change in how people view themselves, others, and the world.

### PTSD, suicidality, shame, guilt, and depression

Last, in a sample of soldiers receiving psychiatric treatment (27), guilt and shame were associated with more severe SI. However, when depressive and PTSD symptoms were controlled for in the analysis, only higher levels of guilt were associated with stronger SI. Also, military personnel who had experienced SI in the past had higher levels of guilt and shame compared to those who had not reported past SI. These results remained significant when the analyses controlled for symptoms of PTSD and depression.

## Discussion

This review investigated the associations between shame, guilt and suicidality in patients who had been diagnosed with PTSD or been exposed to a potentially traumatic event. The association between PTSD and suicidality has already been shown (8) and was again confirmed in several of these studies (27, 29, 30, 33, 35). Associations between shame and guilt and PTSD were demonstrated (27, 29–31), with some studies particularly emphasizing the association between shame and guilt and one or more aspects of suicidality (27, 29–31).

### Involvement of shame and guilt in the suicidality for patients exposed to potentially traumatic events or diagnosed with PTSD

Shame and guilt were found to have direct effects on suicidality in patients who had experienced potentially traumatic events or been diagnosed with PTSD. This involvement was shown in several populations: adolescents (30, 32), women (37), and soldiers (27, 31).

The links between shame, guilt and suicidality in PTSD were not demonstrated in one population: a sample of adolescent girls who had experienced sexual violence (29). Shame and self-blame appeared to be better predictors of SI than PTSD symptoms. PTSD symptoms explained 1.08% of the variance in SI when self-blame, shame, and depression symptoms were included in the model. It should be underlined that these adolescents were closer from the traumatic event compared to the other samples who experienced it much more significantly in the past.

### Influence of the type of traumatic event on SI, shame, and guilt

This review suggests that the effects of shame and guilt on the intensity and frequency of SI vary depending on the type of traumatic event (28, 32).

Emotional abuse, emotional neglect and physical neglect were correlated with shame, whereas sexual and physical abuse weren't. All these abuses, except physical abuse, were correlated with guilt. Physical abuse did not appear to have a direct link with shame and guilt, as the victims tended to become strongly detached, which in the long term increases the risk of aggressive and antisocial behavior (32). Physical abuse, especially when the abuser is a caregiver, results more often in emotional numbing (32).

In a population of soldiers, it appeared that acts of omission were more strongly linked to PTSD, suicide, shame and guilt (31) than were acts of commission. Indeed, large-scale acts of omission were found to have the power to alter world views. These alterations would explain the association of PTSD with suicide more than shame and guilt.

### PTSD, suicidality, shame, guilt, and depression

Results of Sekowski et al. (32) support a model that considers guilt and depressive symptoms as mediating variables in the relationship between sexual abuse or physical neglect and the intensity and severity of SI. In those cases, that involve guilt but not shame, suicidality is a kind of punishment. Moreover, in a sample of soldiers (27), guilt and shame were higher when soldiers had a history of SI, even when depressive symptoms were controlled for. Shame and guilt therefore seem to be major risk factors for SI (27), and these two emotions might therefore be considered risk factors for suicidality in PTSD. Nevertheless, causal attributions (11) (e.g., “it happened because I wasn't paying enough attention”) also contribute to the development of shame and guilt, as well as depression. Thus, an investigation into the psychiatric history and comorbid disorders (17) is essential because suicide is linked to a multifactorial process (26).

### Axis which can be developed in the therapeutic work

Abuse frequently results in dysregulations of emotions and development of inadequate coping strategies (32). In the study of Alix et al. (29), high levels of avoidance were positively associated 6 months later with symptoms of PTSD and SI. As the different models of suicide highlight, however, the perceived inadequacy of coping strategies can be a trigger for SI (16). Therapeutic work on emotions might therefore indirectly reduce SI and PTSD symptoms. For example, it appeared that shame and guilt were associated with feelings of helplessness and negative self-esteem (10), which might well have grown out of the inability to protect oneself from abuse. Therapeutic work on shame should thus address the need to improve self-esteem and self-worth. However, the aggressor's behavior might also influence the development of shame and guilt because the victim has internalized his or her negative remarks and messages (28). In this case, the aggressor might strive to induce shame and guilt as a way of ensuring the victim's silence (28). Silence leads to isolation, yet social support is negatively associated with suicide and helps to regulate negative emotions and cognitions (19, 30). In this case, work on guilt should involve work on social relationships (28).

## Limitations

This review has several limitations. First, only few studies were available for the review, with a limited set of populations. In fact, the associations between shame, guilt, and suicidality in the context of PTSD were to a great extent observed in specific populations already at risk: soldiers (27, 31), borderline personalities (33, 35), patients already diagnosed for a depressive episode (37), and patients hospitalized in a psychiatric unit (28, 32). This specificity makes the results difficult to generalize.

It should be underlined that shame and guilt need to be well-defined to avoid confusion with notions such as self-blame (29, 30). Self-blame is a cognition, which nevertheless might lead to the development of shame. However, shame is believed to be a better predictor of PTSD symptoms than self-blame (30).

Only two longitudinal studies (30, 37) were included in this review. Further longitudinal studies would help to confirm or refute the predictive validity or causal links between shame, guilt and suicidality in patients who have experienced potentially traumatic events or been diagnosed with PTSD.

Furthermore, none of the papers included in this review deal with COVID-19 traumatic experiences. Cavalera (38) underlines that shame and guilt are particularly involved in this context, because of infection or death of relatives, exposition to social medias, and feeling of helplessness. The role of shame and guilt in those experiences and their consequences on the mental and somatic health in the long run should be examine more precisely.

## Conclusion

This is the only systematic review to date that has investigated the implications of shame and guilt in the suicidality in patients who have experienced potentially traumatic events or been diagnosed with PTSD. The articles included were published in the last decade, which points to a new interest in the implication of these emotions in psychiatric disorders, particularly PTSD, and suicidality.

Suicide risk assessment is particularly important for people diagnosed with PTSD or who have experienced a potentially traumatic event. The results of this review show that the emotions of shame and guilt may be risk factors for suicide in these patients. The systematic identification of these two emotions would make it possible to anticipate the development of SI or the passage to a suicidal act, thus ensuring that offers of adapted therapeutic help—for example, therapy based on self-compassion or therapy oriented toward acceptance and commitment—are made in a timely manner.

The studies included in this review showed evidence of the relationships between shame and suicidality and PTSD, and it should be noted here that most of the studies focused specifically on shame. As shame and guilt are correlated (27, 28), it might be worthwhile to examine the implications of guilt in suicidality and PTSD more closely. Interestingly, Bryan et al. (29) measured guilt in soldiers who had been treated for mental health problems and found that the general tendency toward guilt was more strongly related to shame than the feeling of guilt about a specific event. It might therefore be fruitful to compare “trait” or “state” guilt—like “trait” or “state” anxiety—to assess their respective implications in suicidality and PTSD.

## Data availability statement

The original contributions presented in the study are included in the article/Supplementary material, further inquiries can be directed to the corresponding author.

## Author contributions

All authors listed have made a substantial, direct, and intellectual contribution to the work and approved it for publication.

## Conflict of interest

The authors declare that the research was conducted in the absence of any commercial or financial relationships that could be construed as a potential conflict of interest.

## Publisher's note

All claims expressed in this article are solely those of the authors and do not necessarily represent those of their affiliated organizations, or those of the publisher, the editors and the reviewers. Any product that may be evaluated in this article, or claim that may be made by its manufacturer, is not guaranteed or endorsed by the publisher.
