# Supplementary material for: Shame and guilt in the suicidality related to traumatic events: A systematic literature review
Source: Front Psychiatry. 2022 Sep 30;13:951632. doi: 10.3389/fpsyt.2022.951632 (PMC9581050; doi:10.3389/fpsyt.2022.951632)
Supplement: Supplementary file 1 [file Table_1.DOCX]

Table 1. Studies included in the review

| Reference | Sample | Main variables | Secondary variables | Results |
| --- | --- | --- | --- | --- |
| 27 | N=147  Adolescents (14 to 18 yr) having experienced sexual violence | PTSD: CITES II & PCL  Shame: ASSQ  SI in the last 3 months: 1 item of the YSR | Self-blame: AAI  Coping strategies: WCQ  Symptoms of depression: Affective problems scale from the YSR  Description of the abuse: HVF | 66.40% with PTSD; 53% reported depression; 45.80% reported suicidal ideation (SI) in the last 3 months  Self-blame a significant predictive factor for SI (β=0.41; p<0.001), shame (β=0.40; p<0.001), PTSD (β=0.38; p<0.001)  1.08% of the variance of SI explained by symptoms of PTSD when self-blame, shame, and symptoms of depression were included in the model  41.60% of the variance in SI explained by self-blame, shame and depressive symptoms; 45.20% of the variance of PTSD symptoms explained by shame and self-blame  Shame and depression symptoms mediated the relationship between self-blame and SI (z=2.06; p<0.05) |
| 28 | N=100  Adolescents (14 to 18 yr) having experienced sexual violence | Longitudinal study with measures at T0 and T1 (+6 months)  (T1) Description of the abuse (number, severity, duration, relation with the abuser): HVF  (T2) TSPT: CITES II  (T1) Shame: ASSQ  (T2) Suicidal ideation: item 91 of the “Affective Problems Scale” of the YSR | (T1) Coping strategies: WCQ  (T2) Symptoms of depression: YSR  (T1) Self-blame: AAI | 50% had experienced sexual abuse in the preceding month; 17.7% reported abuse more than 18 months before the study; 84.4% had experienced severe sexual abuse (penetration or attempted penetration); 57.1% reported a single episode of abuse  48% reported symptoms of PTSD; 37.4% reported symptoms of depression; 30.3% reported SI  Significant correlations (p<0.05) between SI at T2 and shame (r=0.314), symptoms of PTSD (r=0.358), and symptoms of depression (r=0.573)  54% of the variance of SI at T2 was explained by avoidance at T1 and depressive symptoms at T2  PTSD symptoms did not predict SI when all variables were included in the model (β=0.25; p=0.055)  Shame at T1 was positively correlated with PTSD symptoms at T2 (r=0.444; p<0.05) and SI at T2 (r=0.314; p<0.01)  11% of the variance in PTSD at T2 was explained by shame at T1  Self-blame was the only predictor of symptoms of depression (β=0.22; SE=0.30; p=0.026) |
| 29 | N=69  Soldiers who had been treated for mental health problems  95% were in the Air Force  23.6% had been diagnosed with PTSD  Each soldier had between 1 and 4 symptoms | PSTD: PCL  Shame and guilt: PFQ-2  Past suicidal ideation: SITBI  Current suicidal ideation: BSSI | Symptoms of depression: PHQ-9 | 36.2% of the patients had thought of suicide at least once in their lives; correlations of SI and:   - PTSD: r=0.40; p<0.01 - symptoms of depression: r=0.38; p<0.01 - shame: 0.41; p<0.01 - guilt: r=0.41; p<0.01   Correlation of PTSD and shame: r= 0.44; p<0.01, and guilt: r=0.47; p<0.01  Correlation between shame and guilt: r=0.72; p<0.01  Correlation between depressive symptoms and PTSD: r=0.74; p<0.01  Patients reporting past SI compared to those not reporting it:   - higher level of guilt: M=11.44; SD=4.94 (vs M=6.89; SD=4.91) - higher level of shame: M=17.64; SD=6.75 (vs M=11.57; SD=6.64) - results always significant when PTSD and depression symptoms controlled for   As independent predictors, shame (*B*=0.120; *SE*=.029; p<0.001, OR=1.13 [1.07, 1.19]) and guilt (*B*=0.194; *SE*=.039; p<0.001; OR=1.21 [1.12, 1.31]) were associated with more severe current SI  Guilt associated with current SI was stronger when depressive symptoms and PTSD were controlled for (*B*=0.179; *SE*=.053; p=0.001)  Increase in SI significantly associated with depression symptoms (*B*=0.229; *SE*=0.113; p=0 .043) and PTSD symptoms (*B*=0.082; *SE*=0.030; p=0.006) |
| 30 | N=13  Women diagnosed with PSTD and personality disorder with suicidal behaviors or self-mutilation in the preceding 3 months | PTSD: PSS-I  Shame: ESS  Guilt associated with trauma: TRGI  Self-mutilation and suicidal attempts: SASII  Frequency of suicidal ideation: SBQ | Dissociation: DES  Depression: HRSD  Anxiety: HAM  Social functioning: GSA | 61.5% had been sexually abused in childhood, 23.1% had made an SA within the year, 92.3% had been self-harming; during treatment, 3 self-harmed and 1 made an SA. Effects of the combination of dialectical behavioral therapy (DBT) and prolonged exposure therapy (PE) on:   - PTSD: d=1.7; significant change (powerful effect); 70 to 86% of patients showed a significant and stable reduction in symptoms - SI: d=1.0; significant change; M=10.7 with treatment; M=5 at 3 months post-treatment - shame: d=1.3; significant change (powerful effect) - guilt: d=0.7; non-significant change |
| 31 | N=26  Women diagnosed with PTSD and personality disorder with suicidal behavior or self-mutilation in the preceding 5 years | PTSD: PSS-I  Guilt associated with trauma: TRGI  Shame: ESS  Self-mutilation and suicide attempts: SASII | Dissociation: DES  Psychological well-being: GSI  Depression: HRSD  Anxiety: HAM | Effects on PTSD symptoms:   - with DBT+PE: g=1.4 to 1.6; very broad - with DBT alone: g=0.9; broad   Patients who received DBT+PE were 2.4 times less at risk of SA and 1.5 times less at risk of self-harming behavior  DBT + PE had a very broad effect on dissociation, guilt, shame, anxiety and depression (g=1.2 vs g=0.9 in those with DBT only) |
| 32 | N=50  Soldiers who had served in Afghanistan Irak;  All branches of the Army were represented | PTSD: PCL-5  Shame and guilt: 7 items used in the research of Thompson and Berenbaum, 2006  Suicidality: 4 items of the SBQ-R | Acts of commission and omission: 2 items of the MIES  Violating one’s own moral values: 1 modified item from the MIES  Deployment risk: DRRI-2  Change in perceptions: 6 items of the SLES  Depression: MASQ  Alcohol consumption: AUDIT-C | Partial correlations removed the shared variance with age, sex and pre/post combat experience   - acts of commission and PTSD: r=0.11; ns - acts of omission and PTSD: r=0.38; p<0.05 - shame + guilt and PTSD: r=0.67; p<0.001 - acts of omission and suicidality: r=0.36; p<0.05 - acts of commission and suicidality: r=0.23; ns - suicidality and shame + guilt r=0.53; p<0.001   Altered perceptions mediated the association between inaction and:   - PTSD: 0.37, 95% CI [.17–.58] - suicidality: 0.28, 95% CI [.11–.46]   Correlations between acts of commission and:   - shame and guilt: r=0.42; p<0.01 - altered perceptions: r=0.45; p<0.01   Correlations between acts of omission and:   - shame and guilt: r=0.74; p<0.001, - altered perceptions: r=0.53; p<0.001 |
| 33 | N=112  Adolescents between 12 and 17 years old; hospitalized in a psychiatric unit | Childhood abuse: CTQ  Shame and guilt: PFQ-2  Frequency of SI and suicidal behavior: C-SSRS | Symptoms of depression: BDI-II | 67.9% had major depression; 38.4% had a history of SA, 33.9% of which occurred in the past year  48.2% had suffered some form of abuse, of which 61.1% had experienced moderate or severe abuse  Shame and guilt positively and significantly associated with depressive symptoms (β=0.371; p<0.008 and β=0.335; p<0.001).  Correlation between shame and guilt: r=0.718; p<0.001  Significant correlations (p<0.05) between shame and emotional abuse (r=0.386), emotional neglect (r=0.290), physical neglect (r=0.216); non-significant correlations (p>0.05) between shame and physical abuse (r=-0.026) or sexual abuse (r=0.145)  Significant correlations (p<0.05) between guilt and emotional abuse (r=0.296), emotional neglect (r=0.281), physical neglect (r=0.388), sexual abuse (r=0.189); non-significant correlation (p>0.05) between guilt and physical abuse (r=-0.056).  Significant indirect negative effect of physical abuse on intensity (z=-2.82; p=0.005) and severity (z=-2.78; p=0.006) of SI mediated by guilt and depressive symptoms  Significant indirect positive effect of sexual abuse on intensity (z=2.02; p=0.043) and severity (z=2.03; p=0.042) of SI mediated by guilt and depressive symptoms; indirect non-significant positive effect of sexual abuse on intensity (z=1.47; p=0.143) and severity (z=1.47; p=0.141) of SI mediated by shame and depressive symptoms  Significant indirect positive effect of physical neglect on intensity (z=2.26; p=0.024) and severity (z=2.33; p=0.020) of SI mediated by guilt and depressive symptoms; indirect non-significant positive effect of physical neglect on intensity (z=-0.42; p=0.674) and severity (z=-0.42; p=0.675) of SI mediated by shame and depressive symptoms  Emotional neglect not significantly associated with shame and guilt when other forms of abuse were experienced at the same time: r=0.147; p>0.05 |
| 34 | N=106  Women who had been victims of sexual abuse in childhood and diagnosed with depression (criteria from DSM-IV-R) | Longitudinal study with measures at weeks 0, 10, 24 and 36  Symptoms of PTSD: MPSS-SR  Emotions (e.g., shame and guilt): DES-IV  Suicidal ideation: BDI-II & HRSD  Past suicide attempts: SASII | Symptoms of depression: BDI-II & HRSD | At the start of the study, 46.23% did not have SI; 49.06% had “average” SI (“Feels life is not worth living”) 4.72% had “moderate” (“Wishes she were dead or any thoughts of possible death to self”) SI and 0% had “severe” (“Suicidal ideas or gestures”) ideation No significant change over the 4 measurement times  Depressive symptoms had the greatest influence on multivariate results (93%), followed by history of SA (32%) and PTSD symptoms (18%); age had no influence (0%)  Results of BDI and HRSD, adjusted for sociodemographic variables, symptoms of depression, PTSD symptoms and history of SA (OR; 95% CI): OR (C/score BDI) =1.24; OR(C/HRSD)=1.16; OR (H/score BDI)=1.13; OD(H/score HRSD)=1.02 |
| 35 | N=68  Women who had been admitted to a psychiatric unit; 18 to 61 years old | Sexual abuse in childhood: CTQ  Frequency of affect associated with shame and guilt: PHQ-2  Suicidal ideation: SBQ-R | Symptoms of depression: PHQ-9 | 41% experienced acts of sexual abuse in childhood  Correlations when depressive symptoms were controlled for:   - frequency of SI and shame: r=0.33; p<0.005 - frequency of SI and guilt: r=0.27; p<0.05 - shame and guilt: r=0.63; p<0.01   Negative association between childhood sexual abuse and guilt: t= –2.74; p<0.01  Positive association between childhood sexual abuse and shame: t=2.64; p<0.01  Frequency of SI:   - among women who have never been abused: 3.5 - among women who have been abused: 2.5 |

*Note*. AAI: Abuse Attribution Inventory; ASSQ: Abuse Specific Shame Questionnaire; AUDIT-C: Alcohol Use Disorders Identification Test-Consumption; BDI-II: Beck Depression Inventory II; BSSI: Beck Scale for Suicidal Ideation; CITES II: Children’s Impact of Traumatic Events Scale II; C-SSRS: Columbia Suicide Severity Rating Scale; CTQ: Childhood Trauma Questionnaire; DES: Dissociative Experiences Scale; DES-IV: Differential Emotions Scale-IV; DRRI-2: Deployment Risk and Resilience Inventory-2; ESS: Experience of Shame Scale; GSA: Global Social Adjustment; GSI: Global Severity Index; HAM: Hamilton Anxiety Rating Scale; HRSD: Hamilton Rating Scale for Depression; HVF: History of Victimization Form-French Version; ISS: Internalized Shame Scale; MASQ: Mood and Anxiety Symptoms Questionnaire; MIES: Moral Injury Events Scale; MPSS-SR: Mood and Physical Symptoms Scale-Revised; PAI: Personality Assessment Inventory; PCL-5: PTSD Checklist for DSM-5; PFQ-2: Harder Personal Feelings Questionnaire; PHQ-2: Personal Feelings Questionnaire-2; PHQ-9: Patient Health Questionnaire-9; PSS-I: PTSD Symptom Scale; SASII: Suicide Attempt Self Injury Interview; SBQ: Suicidal Behaviors Questionnaire; SBQ-R: Suicidal Behaviors Questionnaire-Revised; SIDES: Structured Interview for Disorders of Extreme Stress; SITBI: Self-Injurious Thoughts and Behaviors Interview; SLES: Stressful Life Experience Scale; Suicidal Ideation Scale; TRGI: Trauma-Related Guilt Inventory; WCQ: Ways of Coping Questionnaire; YSR: Youth Self-Report
